# Innovative Linear Low Density Polyethylene Nanocomposite Films Reinforced with Organophilic Layered Double Hydroxides: Fabrication, Morphology and Enhanced Multifunctional Properties

**DOI:** 10.1038/s41598-017-18811-y

**Published:** 2018-01-08

**Authors:** Jiazhuo Xie, Haijun Wang, Zhou Wang, Qinghua Zhao, Yuechao Yang, Geoffrey I. N. Waterhouse, Lei Hao, Zihao Xiao, Jing Xu

**Affiliations:** 1 College of Chemistry and Material Science, Shandong Agricultural University, Tai′an, 271000 China; 2 National Engineering Laboratory for Efficient Utilization of Soil and Fertilizer Resources, National Engineering & Technology Research Center for Slow and Controlled Release Fertilizers, College of Resources and Environment, Shandong Agricultural University, Tai′an, 271000 China; 3 State Key Laboratory of Nutrition Resources Integrated Utilization, Shandong Kingenta Ecological Engineering Co., Ltd., Linshu, 276700 China; 4 Department of Basic Courses, Shandong Medicine Technician College, Tai′an, 271000 China; 5 School of Chemical Sciences, The University of Auckland, Auckland, 1142 New Zealand

## Abstract

Herein, we reported the successful development of novel nanocomposite films based on linear low density polyethylene (LLDPE) with enhanced anti-drop, optical, mechanical, thermal and water vapor barrier properties by introducing organophilic layered double hydroxides (OLDHs) nanosheets. OLDHs loadings were varied from 0–6 wt.%. Structural analyses using the Fourier transform infrared spectrum (FT-IR), X-ray diffraction (XRD), transmission electron microscopy (TEM), scanning electron microscopy (SEM) and energy dispersive X-ray spectroscopy (EDX) indicated that the OLDHs nanosheets were homogeneously dispersed with an ordered alignment in the LLDPE matrix. The LLDPE film containing 2 wt.% OLDHs (denoted as OLDHs-2) showed the optimal mechanical, thermal and water vapor barrier properties, whilst the anti-drop and optical performance of the films improved with increasing OLDHs content. The enhanced antidrop properties of the composite films relative to pristine LLDPE can be expected to effectively reduce agricultural losses to disease when the films are applied as agricultural films, whilst the superior light transmittance and water-retaining properties of the composite films will boost agricultural production. Results presented suggest that multifunctional LLDPE/OLDHs nanocomposites show great promise as low cost agricultural plastic films.

## Introduction

Over the past few decades, the application of agricultural plastic films has increased significantly due to their excellent water-retaining and heat-retaining properties, both of which enhance crop growth rates and yields^1–3^. Globally, millions of tons of agricultural plastic films are used each year^4–6^. Among the commercial polymers used as films in the agricultural sector, LLDPE is the most widely used because of its low cost, easy processibility into films and good heat preservation^7–11^. However, the overall performance of LLDPE needs to be optimized further, for example by improving its anti-drop, optical, mechanical, thermal and water vapor barrier properties, to better support agricultural production.

Blending LLDPE with inorganic fillers (e.g., French chalk, metal oxides, silica and China clay) to produce composite films is a commonly used approach for modifying the structural and physical properties of LLDPE^12–15^. However, the generally low compatibility of the inorganic filler with LLDPE matrix can lead to a non-uniform filler distribution and thus optical and structural irregularities in the films, significantly reducing the performance of the composite films in agricultural applications^16,17^. Therefore, considerable research effort is now being directed towards the development of novel low cost inorganic fillers that can significantly enhance the properties and performance of LLDPE-based nanocomposite films^18–20^.

Layered double hydroxides (LDHs) are synthetic clay-like layered materials with the general formula ${[{{\rm{M}}}_{1-{\rm{x}}}^{2+}{{\rm{M}}}_{{\rm{x}}}^{3+}{({\rm{OH}})}_{2}]}^{q+}{({{\rm{A}}}^{n-})}_{{\rm{q}}/{\rm{n}}}.{{\rm{yH}}}_{2}{\rm{O}}$ (where M^2+^ = Mg^2+^, Co^2+^, Ni^2+^, Cu^2+^ or Zn^2+^; M^3+^ = Al^3+^ or Cr^3+^; and A^n−^ are charge balancing anions located between the sheets composed of edge-sharing MO_6_ octahedra)^21–25^. LDHs by nature are hydrophilic. By exchanging the interlayer anions in LDHs with long chain organic anions, OLDHs can be obtained that possess excellent compatibility with polymer matrices such as LLDPE^10,26,27^. Recently, OLDHs had have attracted wide interest in photoluminescence, optical devices, electrochemical sensors and pharmaceutical applications due to their ability to enhance the mechanical and optical and barrier properties of polymer-based nanocomposites^28,29^. These studies provided the motivation to fabricate nanocomposite films based on LLDPE and OLDHs, with the aim of enhancing the anti-drop, optical, mechanical, thermal and water vapor barrier properties of LLDPE.

In this study, a series of innovative LLDPE/OLDHs nanocomposite films with different OLDHs contents (0, 1, 2, 4 and 6 wt.%, which is denoted as OLDHs-0, OLDHs-1, OLDHs-2, OLDHs-4 and OLDHs-6) were successfully fabricated via solution casting method. The OLDHs was prepared by introducing an aliphatic long-chain molecule into the interlayer of LDHs nanosheets to achieve better compatibility with the LLDPE matrix^30,31^. The effect of OLDHs loading on the structure and properties (e. g. anti-drop, optical, mechanical, thermal, and water vapor barrier properties) of LLDPE/OLDHs nanocomposite films were systematically evaluated and discussed.

## Results

### Structural and morphological characterization of the OLDHs and the LLDPE/OLDHs films

#### Fourier transform infrared spectrum analysis

**Figure 1:**
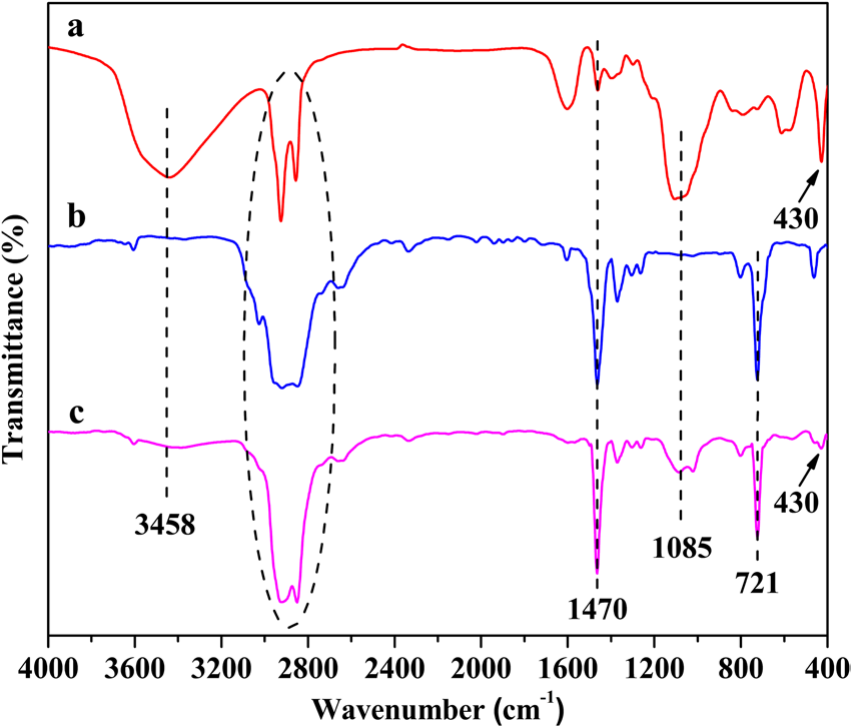
FT-IR spectra for (**a**) OLDHs powder, (**b**) OLDHs-0 and (**c**) OLDHs-6.

The FT-IR, XRD, TEM and SEM were employed to characterize the structural and morphological characterization. The FT-IR spectra of OLDHs powder, OLDHs-0 film and OLDHs-6 film were shown in Fig. 1. The OLDHs powder (Fig. 1a) showed bands at 3458 cm^−1^ and at 430 cm^−1^, which can readily be assigned to O-H stretching and metal-oxygen bending vibrations, respectively, of the LDH sheets. Bands at 2923 cm^−1^, 2853 cm^−1^, 1470 cm^−1^ 1085 cm^−1^ are associated with the organic anions (i.e., C_12_H_25_OPO_3_^2−^ derived from organic modifier, C_12_H_25_OPO_3_K_2_) in the interlayer region, and assigned to C-H stretching (2 modes), CH_2_ scissoring and P-O stretching vibrations, respectively^32^. The data confirms the successful formation of OLDHs. The FT-IR spectrum for the pristine LLDPE films (Fig. 1b) shows bands at 2888 cm^−1^, 1470 cm^−1^ and 721 cm^−1^, due to the characteristic C-H stretching, bending and wagging modes in LLDPE macromolecules, respectively^33^. For the OLDHs-6 film (Fig. 1c), bands associated with the OLDHs and LLDPE were observed, confirming the successful incorporation of OLDHs nanosheets into the LLDPE matrix.

#### X-ray diffraction *analysis*

**Figure 2:**
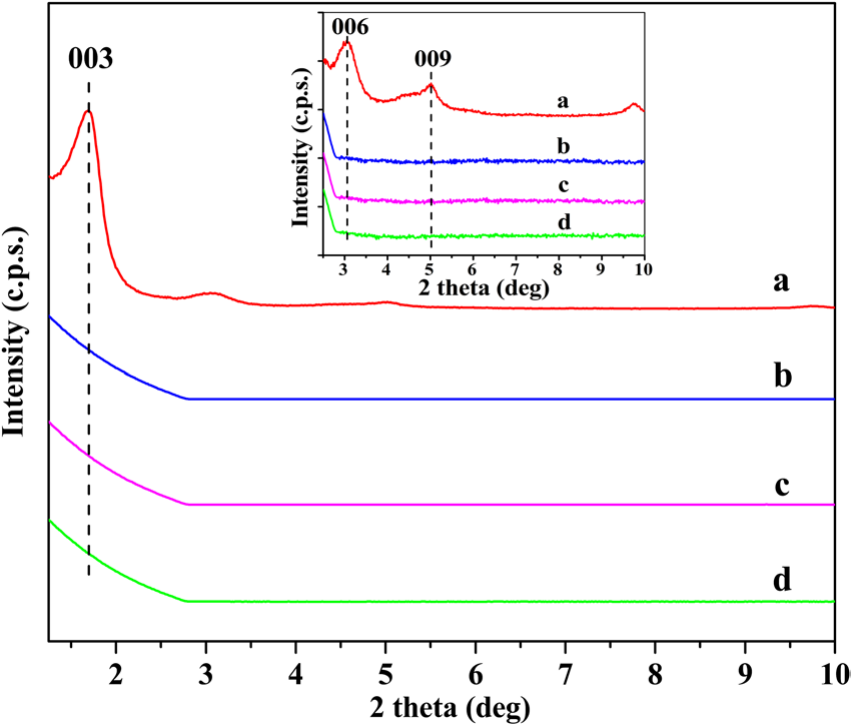
X-ray diffraction patterns for (**a**) OLDHs powder, (**b**) OLDHs-0, (**c**) OLDHs-2 and (**d**) OLDHs-6 in the 2θ range of 1.25–10°.

XRD was also performed to further confirm the structural characterization of the OLDHs powder, OLDHs-0 film, OLDHs-2 film and OLDHs-6 film (Fig. 2). For the OLDHs powder (Fig. 2a), peaks at 1.70, 3.06 and 5.02° can readily be assigned to the (003), (006) and (009) Bragg reflections of a well-developed layered OLDHs structure^34^. The interlamellar spacing of OLDHs was 5.2 nm in our work, while that of inorganic LDHs was 0.79 nm in the previous work^35^. Compared with the LLDPE (Fig. 2b), no LDHs reflections were found in the diffraction patterns for LLDPE films containing OLDHs (Fig. 2c–d). This result suggests that the OLDHs nanosheets had a very uniform distribution in LLDPE matrix. The homogeneous dispersion can be attributed to the easily intercalation of LLDPE macromolecule into the interlamination of OLDHs with lagger interlayer spacing, which can destroy the ordered crystal structure and result in the unordered dispersion of OLDHs nanosheets in the LLDPE matrix. The unordered and uniform dispersion will result in the absence of XRD peaks of OLDHs in the LLDPE/OLDHs films.

#### Morphologies analysis

**Figure 3:**
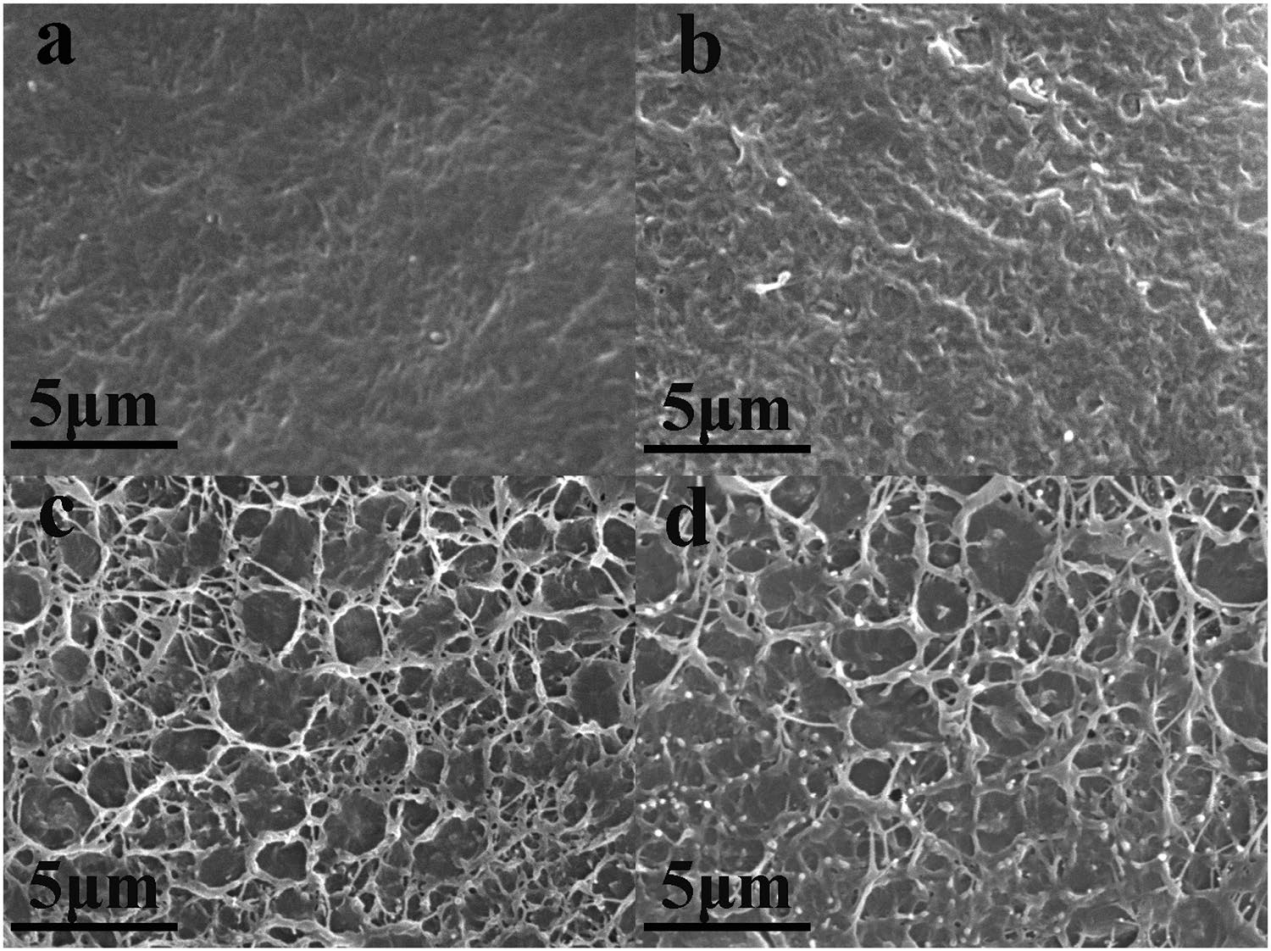
SEM images of the fracture surfaces of (**a**) OLDHs-0, (**b**) OLDHs-1, (**c**) OLDHs-2 and (**d**) OLDHs-6.

TEM and SEM analyses were performed to examine the structure of OLDHs and the dispersion of OLDHs on the fracture surfaces of the pristine LLDPE film (OLDHs-0) and various LLDPE/OLDHs films (OLDHs-1, OLDHs-2 and OLDHs-6). Results are shown in Figs 3, S1 and S2, respectively. From the TEM image, the OLDHs used in our work was a clay-like layered materials in nanoscale with size distribution ~100 nm (Figure S1). The TEM micrograph for the LLDPE/OLDHs film (Figure S2) showed OLDHs nanosheets aligned parallel to the film surface, with this preferential alignment likely resulting from the slow drying process used to fabricate the LLDPE/OLDHs films^36,37^. SEM images of the fracture surfaces of OLDHs-0, OLDHs-1, OLDHs-2 and OLDHs-6 are shown in Fig. 3a–d. It is observed that the OLDHs nanosheets (small white specks in the SEM images) are homogeneously dispersed in the LLDPE nanocomposite at all OLDHs loadings. The fracture surface of OLDHs-0 was quite smooth (Fig. 3a); however the fracture surfaces of the LLDPE/OLDHs films became rougher with OLDHs loading (Fig. 3b–d). The rough fracture surface should be attributed to the adequate interfacial adhesion and compatibility between LLDPE matrix and OLDHs nanosheets. The SEM photomicrographs of OLDHs-2 composites show a more effective nanosheet-matrix adhesion (Fig. 3c), whereas the roughness of the fracture surface was decreased in OLDHs-6 (Fig. 3d). This is a clear indication that at low OLDHs content an effective interaction occurs between the nanosheets and matrix.

#### Elemental analysis

**Figure 4:**
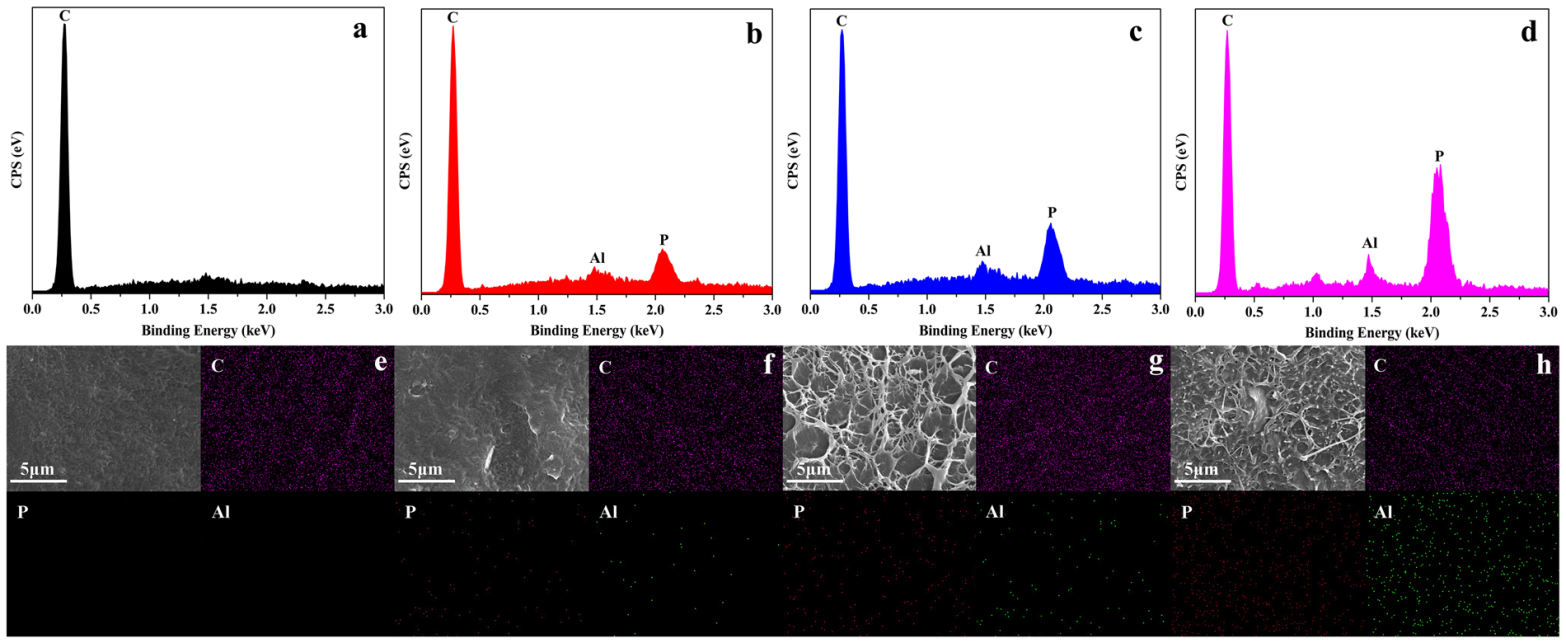
(**a**–**d**) EDX spectra and (**e**–**h**) EDX maps corresponding to the SEM images for fracture surface elemental compositions and distributions of OLDHs-0, OLDHs-1, OLDHs-2 and OLDHs-6, respectively.

To furthermore confirm the dispersion of OLDHs nanosheets, the energy dispersive X-ray spectroscopy detector attached to the scanning electron microscopy (SEM-EDX) were also employed to study the elemental compositions and distributions of the fracture surfaces of LLDPE/OLDHs films. The SEM-EDX spectra (Fig. 4a–d) and maps (Fig. 4e–h) for OLDHs-0, OLDHs-1, OLDHs-2 and OLDHs-6 was shown in Fig. 4, respectively. For the OLDHs-0 film (Fig. 4a,e), only uniformly distributed C element was observed. After the addition of OLDHs nanosheets, the equally distributed P and Al elements appeared on the surface of OLDHs-1 film (Fig. 4b,f). The newly emerging P and Al elements further confirmed the uniform distribution of OLDHs nanosheets in the LLDPE matrix. Moreover, the homogeneous distributions of OLDHs nanosheets in OLDHs-2 and OLDHs-6 films were still retained (Fig. 4c,g and d,h), while more OLDHs nanosheets were added. From the SEM and SEM-EDX results, we can conclude that the OLDHs nanosheets were uniformly distributed in the LLDPE/OLDHs films.

### The effect of OLDHs content to the thermal stability of LLDPE nanocomposite films

**Figure 5:**
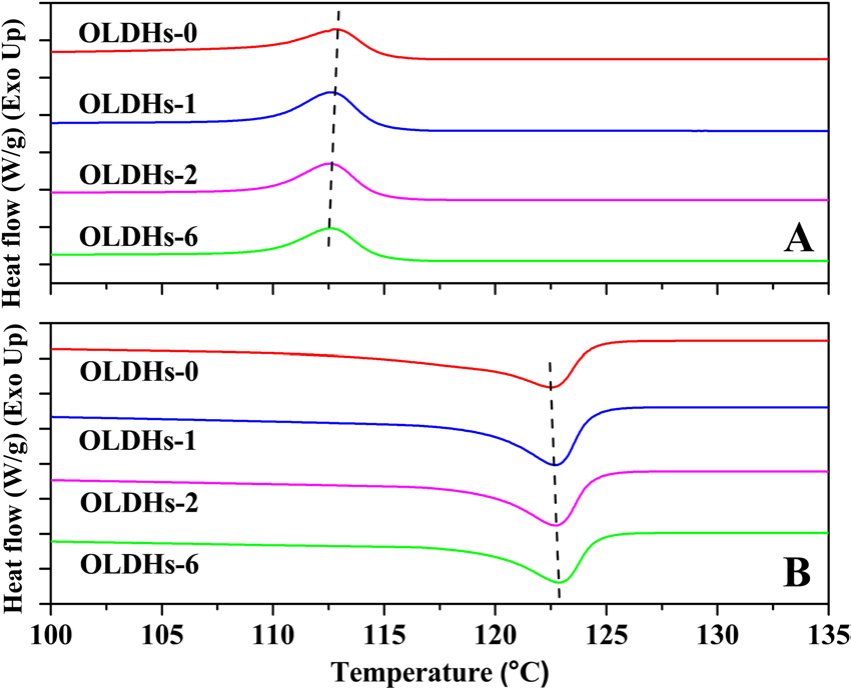
DSC cooling curves (**A**) and heating curves (**B**) for OLDHs powder, OLDHs-0, OLDHs-1, OLDHs-2 and OLDHs-6. The data used were from the first cooling process and the second heating process.

**Figure 6:**
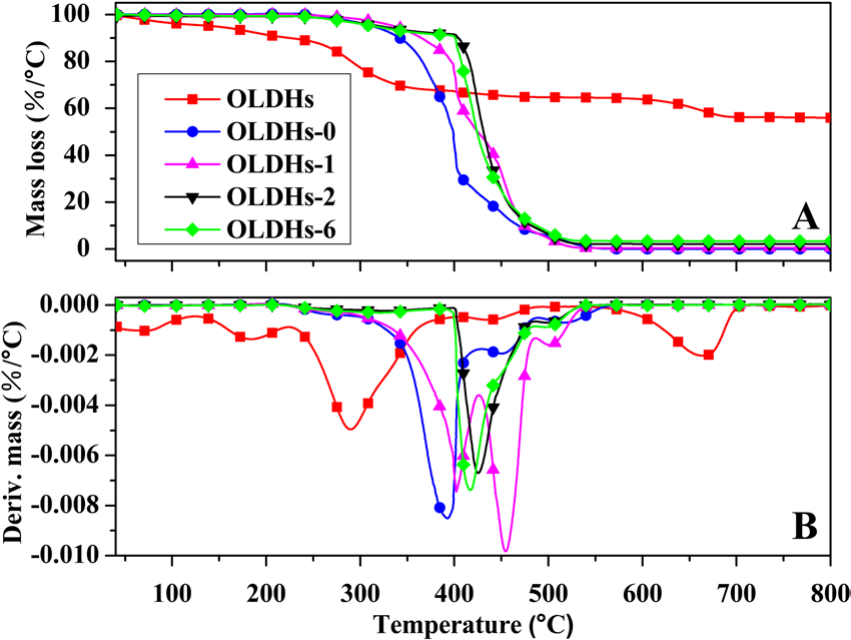
(**A**) TGA and (**B**) DTG curves for OLDHs powder, OLDHs-0, OLDHs-1, OLDHs-2 and OLDHs-6.

Differential scanning calorimeter (DSC) and thermogravimetric analyses (TGA) were employed to study the thermal stability of OLDHs powder, OLDHs-0, OLDHs-1, OLDHs-2 and OLDHs-6 film, which are shown in Figs 5 and 6, respectively. The main findings from the thermal analyses are summarized in Table 1. As shown in Fig. 5 and Table 1, the crystallization temperature (T_c_) of the composite films decreased slightly and the melting temperature (T_m_) of the composite films increased slightly as the OLDHs loading was increased from 0–6 wt.%. These trends with increasing OLDHs loading reflect a decrease in the molecular mobility of the LLDPE chains through the interaction with OLDHs, thereby partially retarding the crystallization and melting of LLDPE chains^36^.

**Table 1:** Thermal analysis data from DSC and TGA curves for LLDPE/OLDHs films.

Sample Films	T_c_ (°C)	T_m_ (°C)	T_10%_ (°C)	T_50%_ (°C)	T_max_ (°C)
OLDHs-0	112.86	122.45	341.87	398.63	391.64
OLDHs-1	112.69	122.62	361.93	424.49	401.35
OLDHs-2	112.53	122.76	399.34	435.18	425.01
OLDHs-6	112.46	122.90	389.64	428.41	416.97

From the thermogravimetric analyses (TGA) and derivative thermogravimetry (DTG) curves for the LLDPE/OLDHs films shown in Fig. 6, it is evident that the thermal stabilities of the LLDPE/OLDHs films increased with OLDHs loading. In general, the temperature at 10% weight loss (T_10%_), the temperature at 50% weight loss (T_50%_) and the temperature of maximum mass loss rate (T_max_) always used to describe thermal stability. In this work, the T_10%_, T_50%_ and T_max_ (Table 1) all increased significantly with the incorporation of OLDHs. The T_10%_, T_50%_ and T_max_ of OLDHs-2 was 54.47 °C, 36.55 °C and 33.37 °C higher than that of pristine LLDPE film respectively. The enhanced thermal stability of OLDHs film (OLDHs-2) was likely due to the adequate interfacial adhesion between OLDHs nanosheets and matrix at low OLDHs content therefore effective hindering heat transfer and thus slowing the decomposition of the LLDPE molecules during the TGA analyse^38^. However, the thermal stability of LLDPE/OLDHs nanocomposite decreased with increasing OLDHs loading (OLDHs-6). One reason for this curious reverse trend was some aggregations occur with inceasing OLDHs loading, which can form heat source domains to accelerate the decomposition of LLDPE matrix in the thermal degradation process. Another reason was that the growing MAPK content in the LLDPE/OLDHs produces less stable charred layers during the decomposition, which would decrease the thermal stabilities of LLDPE/OLDHs nanocomposite films according to the previous work^39,40^. The very similar thermal behaviours have already been reported in some polymer/silicates nanocomposites^41,42^.

### The effect of OLDHs content to the wetability of LLDPE nanocomposite films

**Figure 7:**
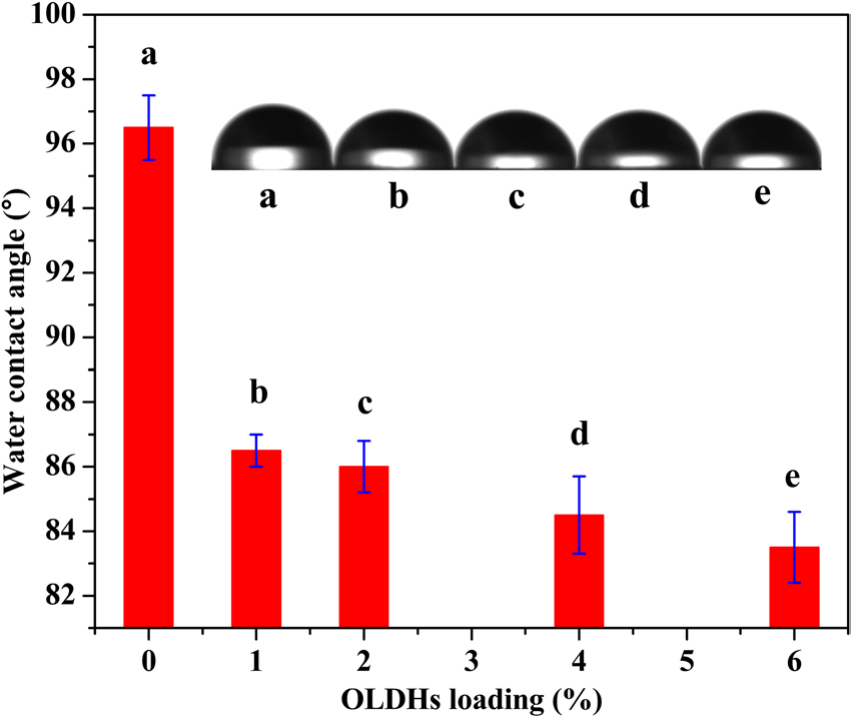
Water contact angles for OLDHs-0, OLDHs-1, OLDHs-2, OLDHs-4 and OLDHs-6.

Water contact angle test was performed to study the wetability of OLDHs-0, OLDHs-1, OLDHs-2, OLDHs-4 and OLDHs-6 film in Fig. 7. As we can see from the results, a decrease in the water contact angle was observed for the LLDPE/OLDHs films as the OLDHs content increased. The water contact angle of OLDHs-6 was 13° lower than that of the pristine LLDPE film (OLDHs-0). The increased hydrophilicity of the LLDPE/OLDHs films can be attributed to the presence of hydrophilic OLDHs nanosheets at the surface of the films^36^. The increased hydrophilicity of the LLDPE/OLDHs films will contribute to increase the water adhesion of the LLDPE films surfaces (i.e., the films will possess better anti-drop properties), to some extent, reducing the water dropped from the films onto the plants and decreasing the production losses to disease.

### The effect of OLDHs content to the optical properties of LLDPE nanocomposite films

**Figure 8:**
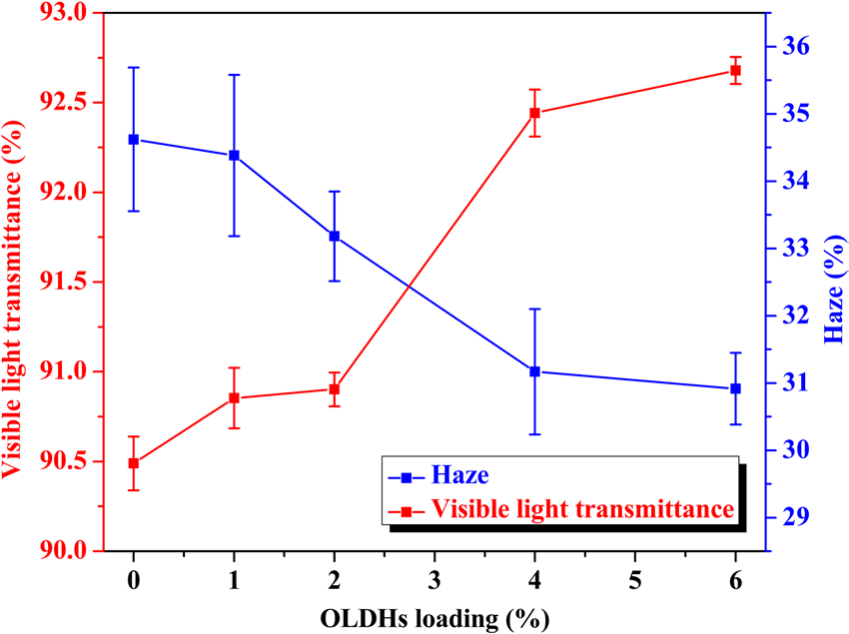
Haze and visible light transmittance of OLDHs-0, OLDHs-1, OLDHs-2, OLDHs-4 and OLDHs-6.

Optical test was employed to study the haze and visible light transmittance of OLDHs-0, OLDHs-1, OLDHs-2, OLDHs-4 and OLDHs-6 in Fig. 8. From the results, the LLDPE/OLDHs films show a visible decrease of haze with increasing OLDHs loading, whilst the visible light transmittance of the films also increased slightly with OLDHs content. The LLDPE/OLDHs film with optimal optical properties was OLDHs-6, showing a 5.84% decrease in haze and 1.39% increase in visible light transmittance compared with the pristine LLDPE film. The improved optical properties can be attributed to heterogeneous nucleation induced by the OLDHs nanosheets in the LLDPE matrix, which has previously been observed in other related studies^43^.

### The effect of OLDHs content to the mechanical performance of LLDPE nanocomposite films

**Figure 9:**
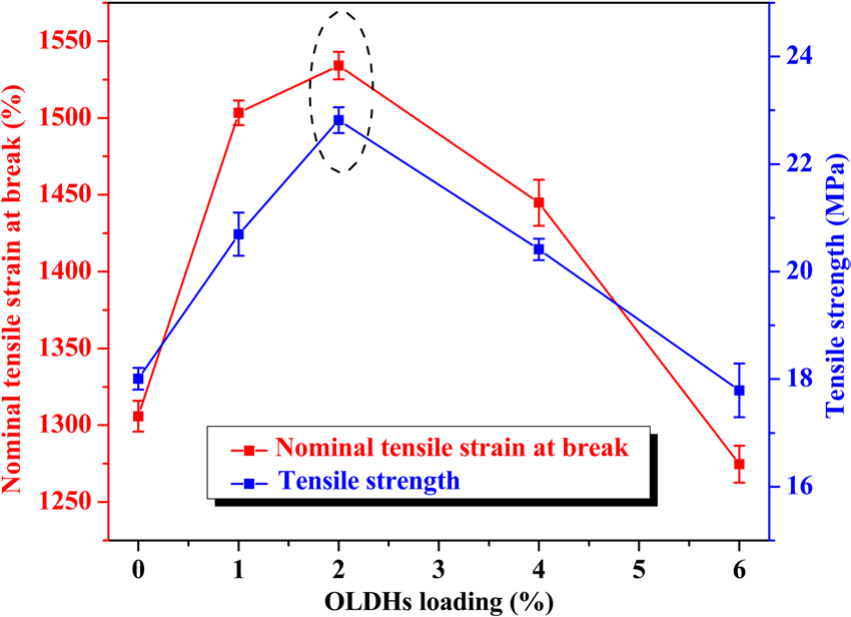
Nominal tensile strain at break and tensile strength of OLDHs-0, OLDHs-1, OLDHs-2, OLDHs-4 and OLDHs-6.

Figure 9 shows the trend of mechanical performances (nominal tensile strain at break and tensile strength) for a pristine LLDPE film and the different LLDPE/OLDHs films. The nominal tensile strain at break and tensile strength of the films increased with OLDHs content up to 2 wt.%, and then decreased sharply at higher OLDHs loadings. OLDHs-2 showed a nominal tensile strain at break and tensile strength of 1534.09% and 22.82 MPa, respectively. These values were 17.48% and 26.71% higher than the corresponding values for pristine LLDPE films. These improvements can be attributed to the uniform dispersion of OLDHs nanosheets in the LLDPE matrix and their intermolecular bonding/entanglement with the LLDPE chains, providing nanoscale junctions to that strengthen the LLDPE matrix. The long-chain anions in the interlayer of the OLDHs promote the formation of interfacial bonds between OLDHs and LLDPE matrix^44^. The decrease of tensile strength with more OLDHs loading (>2%) was attributed to the inevitable aggregation and less adhesion of OLDHs in LLDPE matrix, which will reduce the contact area and intermolecular forces between OLDHs and LLDPE. These support the results of the SEM observations where the OLDHs-2 composites display more effective interaction than the nanocomposites with higher OLDHs content.

### The effect of OLDHs content to the water vapor barrier properties of LLDPE nanocomposite films

**Figure 10:**
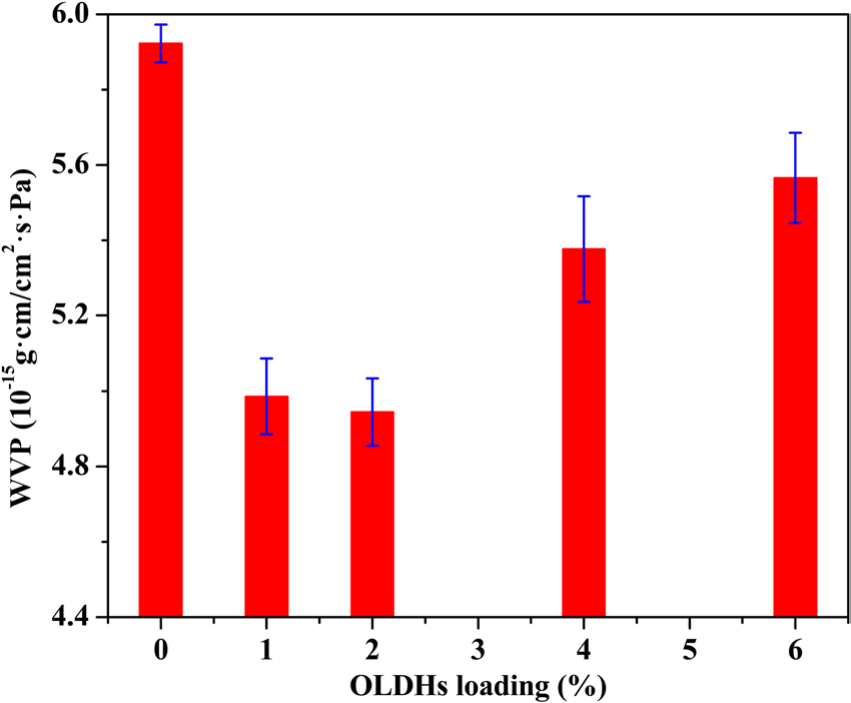
Water vapor permeability curves for OLDHs-0, OLDHs-1, OLDHs-2, OLDHs-4 and OLDHs-6.

The water vapor permeability (WVP) of OLDHs-0, OLDHs-1, OLDHs-2, OLDHs-4 and OLDHs-6 were measured to investigate the influence of OLDHs loading on the water vapor barrier properties of the composite film. Results are shown in Fig. 10. The pristine LLDPE film showed a WVP of 5.92 × 10^−15^ g·cm/cm^2^·s·Pa, suggesting modest water vapour barrier properties. The LLDPE/OLDHs nanocomposite films all possessed lower WVPs and thus superior water vapor barrier properties compared to the pristine LLDPE film. OLDHs-2 showed the lowest WVP value of 4.94 × 10^−15^ g·cm/cm^2^·s·Pa (a 16.55% reduction compared to OLDHs-0). The improvement in the water vapor barrier properties of the films was likely due to the ordered alignment of OLDHs nanosheets in LLDPE matrix, which act as physical barriers to increase the diffusion length of the water molecules as they pass through the film^45^. This has been confirmed by the disappeared OLDHs peaks in the XRD curves (Fig. 2) and the SEM photomicrographs (Fig. 3) of LLDPE/OLDHs nanocomposite (OLDHs-1, OLDHs-2 and OLDHs-6). This pointed out that the addition of a low percentage of OLDHs (OLDHs-2) to LLDPE matrix is sufficient to form a good comparability between them and an improvement in the barrier property of LLDPE/OLDHs composite will occur, as verified in the thermogravimetric analyses tests. Furthermore, the homogeneous dispersity of OLDHs can be visually observed by TEM image (Figure S2) and the elemental analysis (Fig. 4) in the fracture surfaces of LLDPE/OLDHs nanocomposite film. These results suggest that the OLDHs nanosheets had a very uniform distribution in LLDPE matrix. The increasing trend of WVP value with increasing OLDHs loading (4%, 6%) can be attributed to the inevitable aggregation of OLDHs and subsequent less adhesion between OLDHs and LLDPE matrix^46,47^.

## Discussion

Multifunctional agricultural films based on a linear low density polyethylene (LLDPE) matrix modified with organophilic layered double hydroxides (OLDHs) were successfully fabricated. The composite LLDPE/OLDHs films showed enhanced anti-drop, optical, mechanical, thermal and water vapor barrier properties compared with pristine LLDPE films. Considering all these parameters, a composite film prepared with a 2 wt.% OLDHs content demonstrated the best overall performance. The enhanced overall performance can be attributed to a very homogeneous dispersion and ordered alignment of OLDHs nanosheets in the LLDPE matrix. Considering agricultural applications, the enhanced anti-drop properties of the composite films are expected to reduce agricultural losses to disease, whilst the superior light transmittance and water-retention properties of the composite films will enhance agricultural production. Results demonstrate the enornmous potential of low cost LLDPE/OLDHs nanocomposites as multifunctional agricultural plastic films.

## Materials and Methods

LLDPE (trade name: DGM1820) with a melt flow rate (MFR) of 2.65 g/10 min (190 °C, 5.00 kg) was purchased from Sinopec Group (Tianjin, China). NaOH (99%), Zn(NO_3_)_2_·6H_2_O (99%), Al(NO_3_)_3_·9H_2_O (99%), HNO_3_ (65.0–68.0%), toluene (99%) and lauryl phosphoric acid ester potassium (MAPK) (97%) were analytical grade and supplied by Aladdin Reagent Co., Ltd. (Shanghai, China). The deionized water was distilled before used to eliminate dissolved CO_2_.

### Preparation of OLDHs

ZnAl-LDHs precursor was prepared from Zn(NO_3_)_2_·6H_2_O (99%) and Al(NO_3_)_3_·9H_2_O (99%), using a standard two step literature method involving nucleation and aging^48^. An aqueous MAPK solution was prepared by dissolving 0.0275 mol of MAPK in 100 mL of deionized water and adjusting the pH to 4.5 ± 0.1 by the addition of HNO_3_. The OLDHs was prepared from ZnAl-LDHs precursor using an anion-exchange reaction. Briefly, 20 mL of the MAPK solution and 3.0 g of the ZnAl-LDHs precursor were added to a 100 mL three-necked round-bottomed flask. After ultrasonication and subsequent vigorous magnetic stirring under a nitrogen atmosphere for 20 min, the obtained slurry was then aged for 72 h under reflux conditions. The solid product was collected by centrifugation and washed 3 times with deionized water. Finally, the product was dried at 70 °C for 48 h to obtain the white OLDHs powder.

### Fabrication of the LLDPE/OLDHs films

The LLDPE/OLDHs nanocomposite films were fabricated using a solution casting method. Firstly, a certain amount of OLDHs powders, LLDPE granules and 40 mL of toluene were added to a 100 mL three-necked round-bottomed flask. After ultrasonic treatment for 30 min, the mixture was then stirred vigorously at 112 °C under reflux conditions for 8 h. Then, the LLDPE/OLDHs solution was transferred to a glass dish (15 cm × 15 cm × 3 cm) with a flat base. The dish was then placed in a vacuum oven for 6 h at room temperature, after which the temperature was increased to 112 °C over approximately 30 min and then maintained at 112 °C for 6 h under vacuum to obtain the LLDPE/OLDHs films. The total mass of LLDPE and OLDHs was 1.5 g and the thickness of the films was 70 ± 5 μm. The films with the OLDHs contents of 0, 1, 2, 4 and 6 wt.% are denoted as OLDHs-0, OLDHs-1, OLDHs-2, OLDHs-4 and OLDHs-6 in the text below. The fabrication process for the LLDPE/OLDHs composite films is summarized in Figure S2.

### Film structural analysis

FT-IR spectra were collected on a Fourier transform infrared spectrometer (Nicolet 380, Thermo) from 4000 to 400 cm^−1^ at a resolution of 4 cm^−1^ using the KBr pellet method. XRD patterns were recorded from 2θ = 1.25–50° on a X-ray single crystal diffractometer (D8 Quest, Bruker) with a 4°/min scanning speed using a Cu Kα radiation source (λ = 1.54056 Å) operating at a voltage of 40 kV and current of 40 mA. Morphologies of the LLDPE/OLDHs films were examined using a scanning electron microscope (JEOL 6400 F, Japan Electron Optics Ltd.) operating at an accelerating voltage of 5 kV, and a transmission electron microscope (H-800, Hitachi) operating at an accelerating voltage of 200 kV. The elemental analysis was performed at an energy dispersive X-ray spectroscopy (EDX) detector attached to the field emission scanning electron microscopy (SU8010, Hitachi) with an accelerating voltage of 30 kV.

### Film performance tests

The melting and crystallization behavior of the composite films were studied using a differential scanning calorimeter (DSC 200PC, Netzsch). The sample films were first heated to 200 °C from environment temperature and remained at 200 °C for 5 min to eliminate the thermal trace, then cooled to 0 °C and heated to 200 °C again. The heating and cooling rates were 10 °C/min and the sample films mass was 8–10 mg. The data used in our work were from the first cooling process and the second heating process. Thermogravimetric analyses (TGA) and derivative thermogravimetry (DTG) curves were collected using an automatic thermal analysis instrument (DTG-60A, Shimadzu) from 30–600 °C in air, with a heating rate of 10 °C/min. Water contact angles was measured using a contact angle meter (JC2000C2, Zhongchen Digital Technology Instrument Co., Ltd.) by the sessile drop method. Contact angles were determined at five randomly selected points on each film, and the average value calculated. The haze and visible light transmittance of the films were measured with a light transmittance/haze tester (WGT-2S, Yidian Physical Optical Instrument Co., Ltd.) using the GB 2410–2008 standard protocol. Average values of three separate measurements for each film are reported. The nominal tensile strain at break and tensile strength were measured with an electronic universal testing machine (UTM2502, Suns Technology Stock Co., Ltd.) using the GB/T1040.3-2006 standard protocol. Average values of three separate measurements for each film are reported. The water vapor permeability (WVP) of the films was determined using an automatic water vapor transmission tester (PERME W3/030, Instrument Technology Co., Ltd.) using the GB1037-88 standard protocol. The WVP values were calculated using the following equation (1)^49^:

1 $${\rm{WVP}}({\rm{g}}\times {\rm{cm}}/{{\rm{cm}}}^{2}\times {\rm{s}}\times {\rm{Pa}})=({\rm{\Delta }}m\times d)/(A\times t\times {\rm{\Delta }}P)$$

where Δm (g) is the mass loss of water vapor permeating the sample film; d (cm) is the thickness of the sample film; A (cm^2^) is the area of the sample film; t (s) is the measured time interval; ΔP is the difference in water vapor pressure on the two sides of the sample film.

### Data availability statement

The authors declare the data included in manuscript was available.

## Electronic supplementary material

Supplementary Information
