# Supplementary material for: Innovative Linear Low Density Polyethylene Nanocomposite Films Reinforced with Organophilic Layered Double Hydroxides: Fabrication, Morphology and Enhanced Multifunctional Properties
Source: Sci Rep. 2018 Jan 8;8:52. doi: 10.1038/s41598-017-18811-y (PMC5758754; doi:10.1038/s41598-017-18811-y)
Supplement: Supplementary file 1 — Supplementary Information [file 41598_2017_18811_MOESM1_ESM.doc]

**Supporting Information**

**Innovative Linear Low Density Polyethylene Nanocomposite Films Reinforced with Organophilic Layered Double Hydroxides:** **Fabrication, Morphology and Enhanced Multifunctional Properties**

Jiazhuo Xie^1^,^2^, Haijun Wang^1^, Zhou Wang^3^, Qinghua Zhao^4^, Yuechao Yang^2^, Geoffrey I.N. Waterhouse^1,5^, Lei Hao^1^, Zihao Xiao^1^ & Jing Xu^1^

^1^College of Chemistry and Material Science, Shandong Agricultural University, Tai′an 271000, China

^2^National Engineering Laboratory for Efficient Utilization of Soil and Fertilizer Resources, National Engineering & Technology Research Center for Slow and Controlled Release Fertilizers, College of Resources and Environment, Shandong Agricultural University, Tai′an 271000, China

^3^National Engineering Technology Research Center for SCRF, Kingenta Ecological Engineering Group Co., Ltd., Linshu 276700, China

^4^Department of Basic Courses, Shandong Medicine Technician College, Tai′an 271000, China

^5^School of Chemical Sciences, The University of Auckland, Auckland 1142, New Zealand

*Corresponding. [jiaxu@sdau.edu.cn](mailto:jiaxu@sdau.edu.cn)


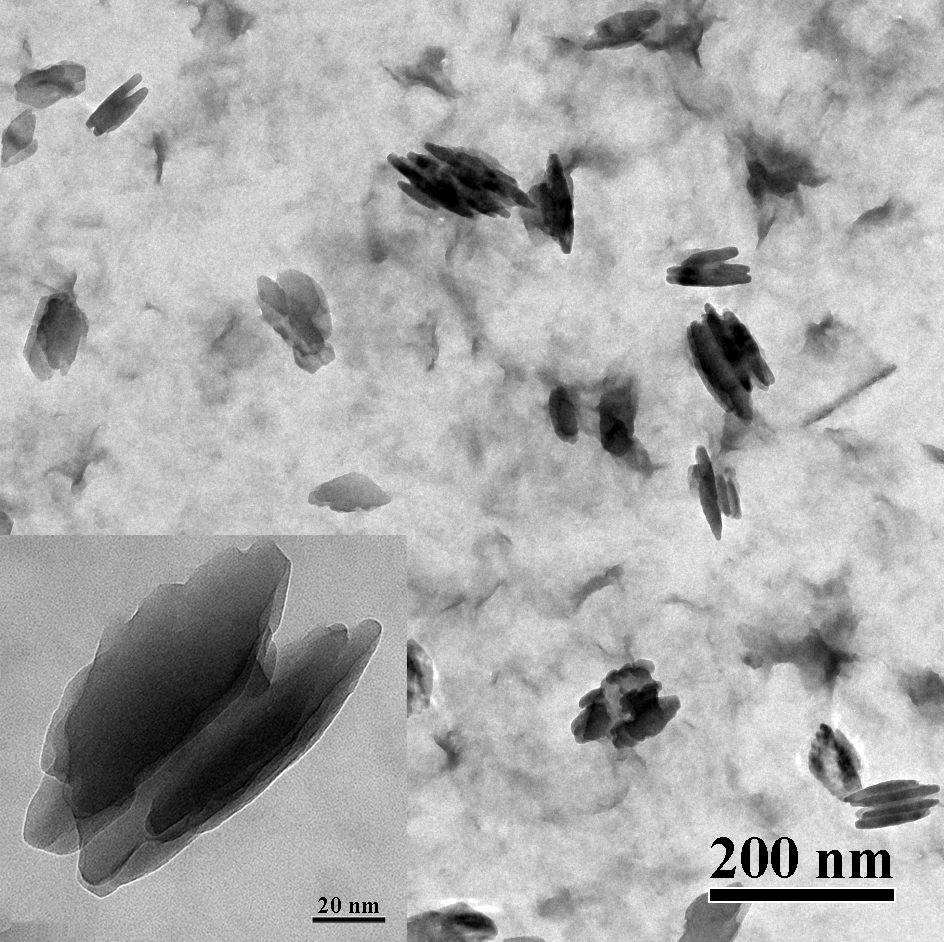


**Figure S1.** TEM image of OLDHs.


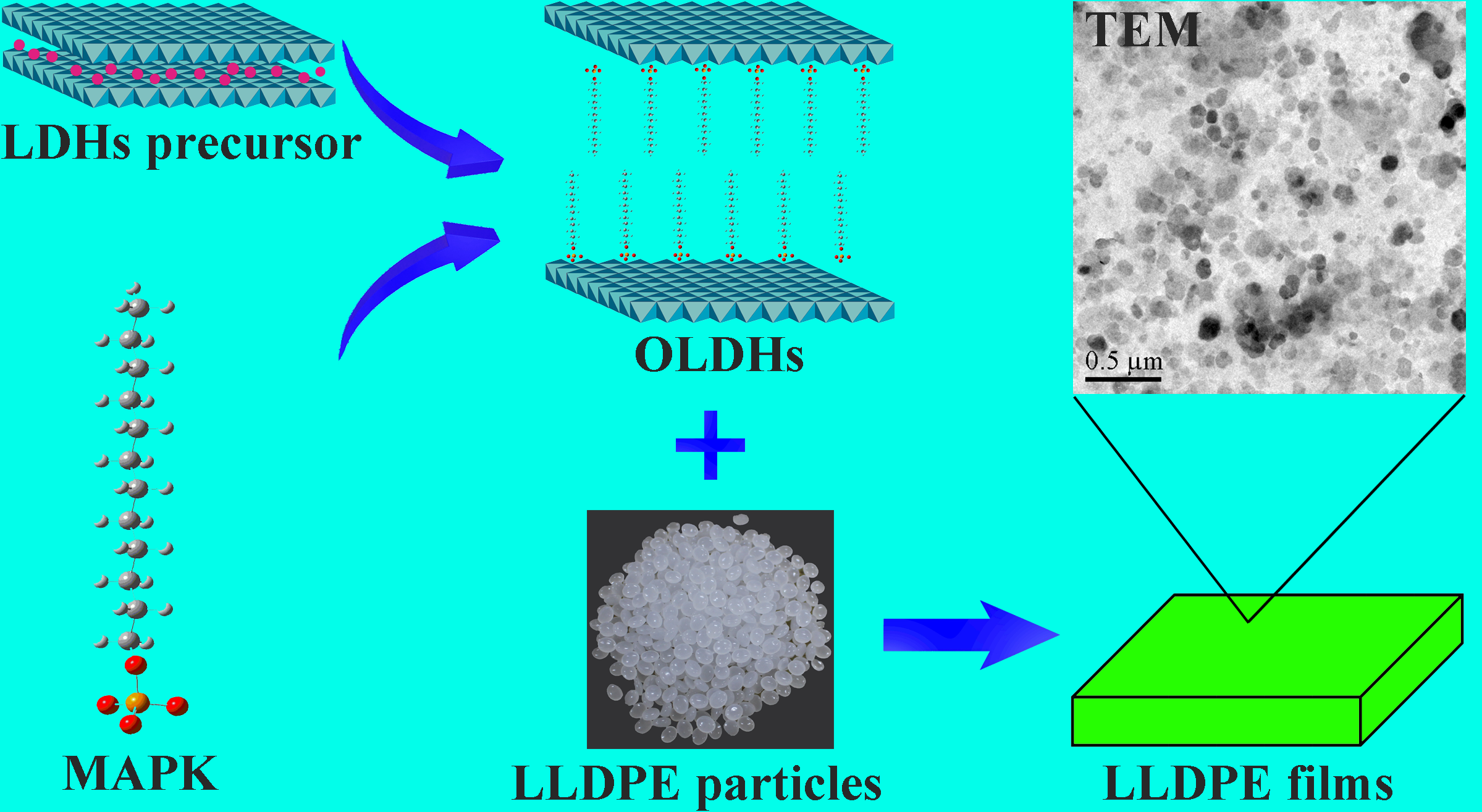


**Figure S2.** Schematic showing the fabrication process of the LLDPE/OLDHs nanocomposite films.
